# Deficiency of the CYLD Impairs Fear Memory of Mice and Disrupts Neuronal Activity and Synaptic Transmission in the Basolateral Amygdala

**DOI:** 10.3389/fncel.2021.740165

**Published:** 2021-09-17

**Authors:** Hui-dong Li, Dan-ni Li, Li Yang, Cheng Long

**Affiliations:** ^1^School of Life Sciences, South China Normal University, Guangzhou, China; ^2^School of Life Sciences, Guangzhou University, Guangzhou, China; ^3^South China Normal University-Panyu Central Hospital Joint Laboratory of Translational Medical Research, Panyu Central Hospital, Guangzhou, China

**Keywords:** cylindromatosis, deubiquitinase, fear memory, basolateral amygdala, neuronal activation, neuronal excitability, synaptic transmission

## Abstract

Fear learning and memory are crucial for animal survival. Abnormal fear memory is a hallmark of many neuropsychiatric disorders. Appropriate neuronal activation and excitability in the basolateral amygdala (BLA) are necessary for the formation of fear memory. The gene *cylindromatosis* (*Cyld*), which encodes a lysine-63 deubiquitinase, is expressed in several brain regions including the amygdala. The functions of the *cylindromatosis* protein (CYLD) in the regulation of the neuronal activity, neural circuits and fear memory, remain largely unknown, however. Here, we report that *Cyld* knockout impairs amygdala-dependent tone-cued fear memory. The number of c-Fos^+^ neurons responding to the tone-cued fear test was reduced in the BLA of *Cyld*^–/–^ mice, suggesting that the absence of CYLD causes aberrant neuronal activation. We found that this aberrant neuronal activation in the BLA of *Cyld*^–/–^ mice may relate to the decreased excitability of principal neurons. Another possibility of aberrant neuronal activation could be the impaired excitatory synaptic transmission in the BLA of *Cyld*^–/–^ mice. Specifically, both the frequency of spontaneous excitatory postsynaptic currents and the amplitude of miniature excitatory postsynaptic currents in BLA principal neurons were decreased. In addition, *Cyld* mutation caused an increase in both the frequency of miniature inhibitory postsynaptic currents in principal neurons and the number of parvalbumin^+^ interneurons, consistent with excessive local circuit inhibition in the BLA of *Cyld*^–/–^ mice. Taken together, these results suggest that CYLD deficiency disrupts the neuronal activity and synaptic transmission in the BLA of mice which may contribute to the impaired fear memory observed in *Cyld*^–/–^ mice.

## HIGHLIGHTS

- *Cyld* knockout impairs amygdala-dependent tone-cued fear memory in mice.

- *Cyld*^–/–^ mice display aberrant neuronal activation in BLA in response to tone-cued fear test.

- *Cyld*^–/–^ mice show decreased excitability of BLA principal neurons.

- CYLD is critical for both excitatory and inhibitory synaptic neurotransmission in mouse BLA.

## Introduction

Learning to identify and respond to threats in the environment is essential for animal survival. Abnormal fear memory is a hallmark of many neuropsychiatric disorders, including Alzheimer’s disease (Varinthra et al., 2021), obsessive-compulsive disorder (Nielen et al., 2009), posttraumatic stress disorder (Bowers and Ressler, 2015), and drug addiction (Taylor and Torregrossa, 2015). In mammals, it is well established that the basolateral amygdala (BLA) plays a central role in the formation and retention of fear memory (Davis and Whalen, 2001; Janak and Tye, 2015; Marek et al., 2019). This form of learning can be achieved with classical auditory fear conditioning by pairing a neutral auditory stimulus conditioned stimulus (CS) with an aversive footshock unconditioned stimulus (US) (Duvarci and Pare, 2014). Broadly speaking, the BLA receives two streams of auditory innervation from the auditory thalamus and auditory cortex and sends projections to the central amygdala (CeA) and eventually to the brainstem and hypothalamus to elicit defensive behavior (freezing) and autonomic and endocrine responses (Johansen et al., 2011).

The BLA forms a cortical-like temporal lobe structure that contains around 85–90% excitatory pyramidal neurons (principal neurons) and 10–15% GABAergic interneurons (Shao et al., 2019). A subset of excitatory neurons can be activated by increased expression of the immediate early gene, *c-Fos* (Tronson et al., 2012). Defective neuronal activation in the BLA of mice subjected to behavioral stimulation is associated with impaired fear memory (Huang et al., 2014). In addition, numerous studies have revealed a crucial role for the excitation of BLA principal neurons in fear memory (Yiu et al., 2014; Polepalli et al., 2020). However, the underlying mechanisms by which neuronal activation and excitability in the BLA are regulated remain elusive.

Cylindromatosis is a tumor-suppressor protein whose gene is mutated in familial cylindromatosis; it serves as a negative regulator of NF-κB signaling (Ma et al., 2017) and is involved in the regulation of important physiological processes such as the immune response, cell cycle, and cell migration (Li et al., 2019). The BioGPS database^1^ shows that *Cyld* (NCBI gene ID: 74256) is highly expressed in the striatum followed by the nucleus accumbens (NAc), amygdala, hypothalamus, prefrontal cortex (PFC), and hippocampus (Probeset numbers: 1429201 and 1429618). Interestingly, according to the *in situ* hybridization data of the Allen Brain Atlas database (experiment 73992938), the protein CYLD mainly distributes in the posterior portion of the BLA^2^. Recent studies have identified CYLD as a protein component of purified rodent postsynaptic density (PSD) (Ma et al., 2017) and a regulator of neuronal cell death (Ganjam et al., 2018), neuronal dendrite morphogenesis, and spine formation (Li et al., 2019). CYLD in the PSD is recruited and further activated in a Ca^2+^-dependent manner by Ca^2+^/calmodulin-dependent protein kinase II to the PSD following high K^+^ or N-methyl-D-aspartic acid (NMDA) stimulation (Zajicek and Yao, 2021). According to recent reports, *Cyld* is a causative gene for frontotemporal dementia (FTD) and amyotrophic lateral sclerosis (Dobson-Stone et al., 2020), and patients carrying *Cyld* variants show severe memory loss (Tábuas-Pereira et al., 2020). Several studies have shed light on the role of deubiquitinases (DUBs), such as ubiquitin-specific protease (USP) and ubiquitin carboxyl-terminal hydrolase L1 (UCHL1), in the modulation of synaptic plasticity and memory processes (Imai et al., 2013; Jarome et al., 2013; Zhang et al., 2014; Zeng et al., 2019; Srikanta et al., 2021). Nevertheless, the functions of CYLD in neuronal activity and neural circuits remain largely unknown.

In this study, by using a combination of electrophysiological, immunohistochemistry, and behavioral measurements, we reveal that *Cyld*^–/–^ mice exhibit abnormal amygdala-dependent tone-cued fear memory. In addition, *Cyld*^–/–^ mice also display aberrant neuronal activation and a decreased excitability of excitatory pyramidal neurons in BLA. What is more, *Cyld*^–/–^ mice exhibit a decrease in both the frequency of spontaneous excitatory postsynaptic currents (sEPSCs) and the amplitude of miniature excitatory postsynaptic currents (mEPSCs) of principal neurons, but an increase in the frequency of miniature inhibitory postsynaptic currents (mIPSCs) and the number of parvalbumin^+^ (PV^+^) interneurons in the BLA. Collectively, these findings suggest that CYLD plays an important role in the regulation of fear memory and acts as a regulator of neuronal activation, neuronal excitability, synaptic transmission and the number of PV^+^ interneuron in the BLA of mice.

## Materials and Methods

### Animals

*Cylindromatosis* knockout mice were initially generously gifted by Dr. Shao-cong Sun (University of Texas MD Anderson Cancer Center, Houston, TX, United States). *Cyld*^–/–^ and *Cyld*^+/+^ mice were generated by intercrossing *Cyld*^+/–^ males and females, and their genotyping was performed as described previously (Zhang et al., 2016). Animal experiments were approved by the Ethics Committee of Animal Research of South China Normal University, following the National Institutes of Health Guidelines for the Care and Use of Laboratory Animals. All procedures were undertaken so as to minimize animal suffering and the number of animals used. All animals were group housed (maximum of five mice per cage) under standard laboratory conditions (24 ± 1°C, 55% ± 5% humidity) and were maintained on a 12:12-h light–dark cycle, with food and water provided *ad libitum*. Two- to 3-month-old male and female mice were used for the experiments.

### Tone-Cued Fear Conditioning

Tone-cued fear conditioning (TFC) was tested using the Panlab Startle and Fear Combined System, which comprised a LE116 experimental chamber, LE1188 stimuli interface unit, LE111 load cell amplifier, and LE10026 shock generator with scrambler (Harvard Apparatus, United States). Two different contexts, context A (metal grid floor, gray chamber walls, and mild alcohol scent) and context B (covered metal grid floor, white chamber walls, and mild acid scent), were used in the TFC experiment. During the training period, each mouse was placed in a chamber. The threshold was set at 2 throughout the experiment based on the sensitive weight transducer of the experimental chamber. On the first day, mice were placed in context A, and after a 3-min habituation stage, they received eight tone (CS)–shock (US) pairings with a 20-s intertrial interval. The shock (0.5 mA, 2 s) was delivered 18 s after the start of the tone (80 dB, 2 kHz, 20 s). Each mouse remained in the same chamber for 2 min before being returned to the home cage. The percentage of freezing time (Freezing %) during the first 3 min in the chamber and each tone-present stage were defined as the habituation freezing level and the learned freezing level, respectively. On the second day, mice were placed in context B for 3 min, and subsequently, we presented a 3-min tone identical to that heard on the previous day, but did not deliver a foot shock (tone-cued test). The Freezing % during the first 3 min and the second 3 min in the chamber was defined as the pre-tone and tone-cued test freezing level, respectively. Freezing % was equal to (freezing time/total time) × 100%. All procedures were coordinated *via* PACKWIN (Harvard Apparatus, United States) on a computer connected to the device, and data were analyzed using the same software.

### Immunohistochemistry

As previously described (Han et al., 2020), mice were anesthetized and perfused transcardially with 4% paraformaldehyde (PFA) in phosphate buffer saline (PBS), and tissues were fixed in 4% PFA at 4°C for 12 h. After dehydration by 30% sucrose, brain tissue was cut into 30-μm-thick sections using a freezing microtome (Leica CM30505, Germany). The posterior portion of the BLA-containing sections was permeabilized with 0.3% Triton X-100 and 5% donkey serum in PBS for 2 h and then incubated with rabbit polyclonal anti-c-Fos antibody (1:1,000, Abcam, Cambridge, United Kingdom, ab190289) or rabbit polyclonal anti-PV antibody (1:1,000, Abcam, ab11427) at 4°C overnight. After washing three times with PBS, sections were incubated with Cy3-labeled anti-rabbit secondary antibody (1:1,000, Absin, Shanghai, China, abs20024) for 1.5 h at room temperature and then given three 10-min washes in PBS. Afterward, sections were incubated with DAPI solution (1:5 for 15 min, Beyotime, Shanghai, China, C1005) for nuclear labeling and coverslipped with anti-fade mounting medium. Images were captured using an EVOS fluorescence microscope (Invitrogen, United States). For c-Fos immunostaining, mice subjected to several behavioral tests were perfused 1.5 h after the tests.

### Whole-Cell Patch-Clamp Recordings

A vibratome (Leica VT1000S, Germany) was used to cut BLA-containing coronal brain slices (thickness: 320 μm) in ice-cold N-methyl-D-glucamine (NMDG)-based cutting solution comprising 93 mM NMDG, 2.5 mM KCl, 30 mM NaHCO_3_, 1.2 mM NaH_2_PO_4_, 20 mM HEPES, 25 mM D-glucose, 2 mM thiourea, 5 mM sodium ascorbate, 3 mM sodium pyruvate, 10 mM MgSO_4_, and 0.5 mM CaCl_2_ (Xing et al., 2021). Brain slices were then transferred to a holding chamber containing artificial cerebrospinal fluid (ACSF) composed of 92 mM NaCl, 2.5 mM KCl, 1.2 mM NaH_2_PO_4_, 30 mM NaHCO_3_, 20 mM HEPES, 25 mM D-glucose, 2 mM thiourea, 5 mM sodium ascorbate, 3 mM sodium pyruvate, 2 mM MgSO_4_, and 2 mM CaCl_2_ (Feng et al., 2021) to recover for at least 1 h at room temperature before being transferred to a recording chamber continually perfused (∼2 ml/min) with oxygenated ACSF (124 mM NaCl, 2.5 mM KCl, 1.2 mM NaH_2_PO_4_, 24 mM NaHCO_3_, 5 mM HEPES, 12.5 mM D-glucose, 2 mM MgSO_4_, and 2 mM CaCl_2_ (Feng et al., 2021). All solutions were bubbled with 95% O_2_/5% CO_2_ throughout the experiment. All recordings were made with 5–6 MΩ pipettes, a MultiClamp 700B amplifier, and a 1440A digitizer (Molecular Devices, United States). Internal solutions for measuring action potential (AP), rheobase, and sEPSCs contained 110 mM K-gluconic acid, 10 mM NaCl, 1 mM MgCl_2_⋅6H_2_O, 10 mM EGTA, 40 mM HEPES, 2 mM Mg-ATP, and 0.3 mM Na-GTP, pH 7.4 (300 mOsm), and for mEPSCs and mIPSCs, the solutions contained 100 mM Cs-methanesulfonate, 10 mM NaCl, 10 mM TEA-Cl, 1 mM MgCl_2_⋅6H_2_O, 10 mM EGTA, 40 mM HEPES, 2 mM Mg-ATP, 0.3 mM Na-GTP, and 4 mM QX-314, pH 7.40 (300 mOsm) (Chen et al., 2020, 2021).

All recordings were performed at room temperature. Principal neurons in the posterior portion of the BLA, which were easily identified morphologically with bright pyramidal-shaped soma under a phase contrast microscope (Xu et al., 2009; Butler et al., 2018; Sun et al., 2018), were chosen to record the AP or postsynaptic currents. For AP recording of excitatory pyramidal neurons in the BLA, pyramidal neurons were held in current-clamp mode and given a series of current pulses (from -120 to 260 pA in 20 pA steps) to elicit APs. Membrane input resistance was calculated in response to a series of hyperpolarizing pulses. For the rheobase test, a ramp current stimulus (from 0 to 200 pA) was given within 500 ms to the principal neurons in the BLA in current-clamp mode. Only experiments with low series resistances (<30 MΩ), stable (<20% variation), and normal membrane potentials (more negative than −60 mV) were analyzed. For sEPSC and mEPSC recording, neurons were clamped at −60 mV, while neurons were clamped at 0 mV during mIPSC recording. mEPSCs and mIPSCs were all recorded in the presence of 300 nM TTX. AP and sEPSCs were measured in the absence of any neurotransmitter blockers. Extra bicuculline (10 μM) or CNQX (20 μM) and APV (50 μM) were added to record mEPSCs or mIPSCs. Data were filtered during acquisition with a low-pass filter of 2 kHz using pClamp 10 (Molecular Devices, United States) and were analyzed offline with Clampfit 10.6 (Molecular Devices, United States).

### Statistical Analysis

Statistical analysis was performed using GraphPad Prism 8 software using either Student’s *t*-test for two-group comparisons or two-way ANOVA with Sidak’s test for multiple comparisons. All data were presented as mean ± standard error of the mean (mean ± SEM). Statistically significant results are indicated either as ^∗^ (*p* < 0.05), ^∗∗^ (*p* < 0.01), or ^∗∗∗^ (*p* < 0.001).

## Results

### Impaired Fear Memory in *Cyld*^–/–^ Mice

**FIGURE 1:**
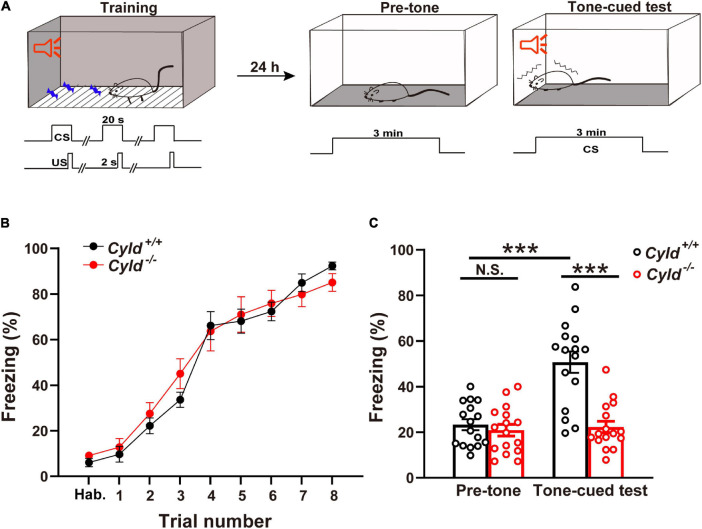
*Cyld* knockout mice show impairment of tone-cued fear-conditioned memory. **(A)** Tone-cued fear conditioning diagram. Tone (CS)–shock (US) pairings were delivered eight times (US, 0.5 mA, 2 s) 18 s after the start of the tone during training in context A. Twenty-four hours later, mice were placed in context B for 3 min (pre-tone) and subsequently subjected to the 3-min tone-cued test in a different chamber. **(B)** *Cyld* knockout did not affect the acquisition phase of fear memory. *Cyld*^–/–^ and *Cyld*^+/+^ mice showed a similar freezing response during the habituation (Hab.) and training period. **(C)** The freezing response of *Cyld*^–/–^ mice was reduced in the tone-cued test. For each group, *n* = 16 mice. Two-way ANOVA, ***p < 0.001; N.S., not significant.

It is reported that patients carrying *Cyld* variants show severe memory loss (Tábuas-Pereira et al., 2020). In view of the certain expression level of CYLD in amygdala (see text footnote 1), we hypothesized that CYLD plays a crucial role in regulating emotional memory. To verify this hypothesis, *Cyld*^+/+^ and *Cyld*^–/–^ mice were subjected to the TFC test (Figure 1A). All mice showed a similar response to the presentation of the tone during the training period [Figure 1B; two-way ANOVA, *F*_(1,30)_ = 0.150, *p* = 0.7012], but 24 h after training, the freezing time of *Cyld*^–/–^ mice in response to the tone-cued test decreased, while *Cyld*^+/+^ mice maintained a relatively high level of fear memory [Figure 1C; two-way ANOVA_(genotype effect)_: *F*_(1,60)_ = 23.39, *p* < 0.0001; two-way ANOVA_(period effect)_: *F*_(1,60)_ = 20.45, *p* < 0.0001; two-way ANOVA_(interaction effect)_: *F*_(1,60)_ = 16.69, *p* = 0.0001; Sidak’s *post hoc* test: pre-tone, *Cyld*^+/+^ vs. pre-tone, *Cyld*^–/–^ *p* = 0.9957; pre-tone, *Cyld*^+/+^ vs. tone-cued test, *Cyld*^+/+^ *p* < 0.0001; tone-cued test, *Cyld*^+/+^ vs. tone-cued test, *Cyld*^–/–^ *p* < 0.0001]. These results suggest that *Cyld* mutant mice experience impaired fear memory.

### Aberrant Neuronal Activation in the Basolateral Amygdala of *Cyld*^–/–^ Mice

**FIGURE 2:**
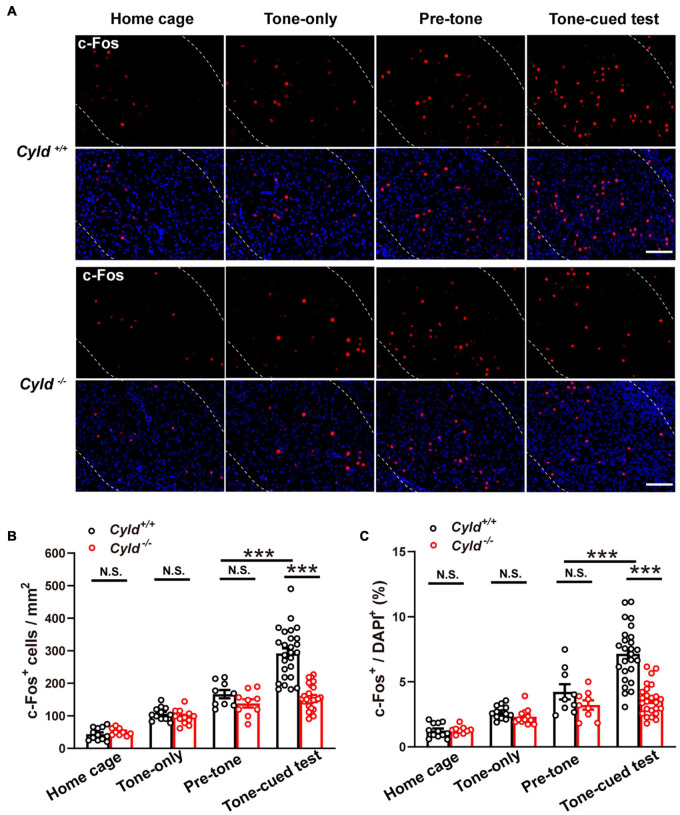
Aberrant neuronal activation in the BLA of *Cyld*^–/–^ mice. **(A)** c-Fos expression in the BLA of the four groups of mice with different experiences: home cage, tone-only, after pre-tone stage, and after tone-cued test. Scale bar: 100 μm. **(B,C)** Quantitative analysis of the c-Fos^+^ neuron number and the percentage of c-Fos^+^ cells in total DAPI^+^ cells in BLA. Home cage: *n* = 12 slices from four mice for the *Cyld*^+/+^ group, *n* = 9 slices from three mice for the *Cyld*^–/–^ group; tone-only: *n* = 12 slices from four mice for the *Cyld*^+/+^ group, *n* = 12 slices from four mice for the *Cyld*^–/–^ group; pre-tone: *n* = 9 slices from three mice for the *Cyld*^+/+^ group, *n* = 9 slices from three mice for the *Cyld*^–/–^ group; tone-cued test: *n* = 26 slices from eight mice for the *Cyld*^+/+^ group, *n* = 25 slices from seven mice for the *Cyld*^–/–^ group; two-way ANOVA, ***p < 0.001; N.S., not significant. Data are presented as mean ± SEM.

The early gene c-Fos is a high-resolution marker of neural activity (Luyck et al., 2020), and numbers of c-Fos^+^ neurons increase during the response to CS–US pairing (Tronson et al., 2012). Fear memory impairment in *Cyld*^–/–^ mice could be due to aberrant neuronal activation in the amygdala (Sun et al., 2018). To test this possibility, we labeled c-Fos^+^ neurons in the BLA of the following four groups of mice with different experiences: home cage, tone-only (mice experienced the 2-day TFC test; but during the training period, the CS was not paired with footshock), after pre-tone stage, and after tone-cued test (Figure 2A). The number of c-Fos^+^ neurons in BLA of *Cyld*^+/+^ and *Cyld*^–/–^ mice was comparable in home cage mice, as well as mice subjected to tone-only or after pre-tone, while the number of c-Fos^+^ cells in response to the tone-cued test was significantly fewer in the mutant than in *Cyld*^+/+^ mice [Figure 2B; two-way ANOVA_(genotype effect)_: *F*_(1,106)_ = 19.98, *p* < 0.0001; two-way ANOVA_(experience effect)_: *F*_(3,106)_ = 87.02, *p* < 0.0001; Sidak’s *post hoc* test: home cage, *Cyld*^+/+^ vs. *Cyld*^–/–^ *p* > 0.9999; tone-only, *Cyld*^+/+^ vs. *Cyld*^–/–^ *p* > 0.9999; pre-tone, *Cyld*^+/+^ vs. *Cyld*^–/–^ *p* = 0.9975; tone-cued test, *Cyld*^+/+^ vs. *Cyld*^–/–^ *p* < 0.0001; pre-tone, *Cyld*^+/+^ vs. tone-cued test, *Cyld*^+/+^ *p* < 0.0001]. Similar results were found when we determined the c-Fos^+^ cells with respect to total cells (DAPI^+^ cells) in the BLA [Figure 2C; two-way ANOVA_(genotype effect)_: *F*_(1,106)_ = 19.41, *p* < 0.0001; two-way ANOVA_(experience effect)_: *F*_(3,106)_ = 55.27, *p* < 0.0001; Sidak’s *post hoc* test: home cage, *Cyld*^+/+^ vs. *Cyld*^–/–^ *p* > 0.9999; tone-only, *Cyld*^+/+^ vs. *Cyld*^–/–^ *p* > 0.9999; pre-tone, *Cyld*^+/+^ vs. *Cyld*^–/–^ *p* = 0.9662; tone-cued test, *Cyld*^+/+^ vs. *Cyld*^–/–^ *p* < 0.0001; pre-tone, *Cyld*^+/+^ vs. tone-cued test, *Cyld*^+/+^ *p* < 0.0001]. These results suggest that aberrant neuronal activation occurs in the BLA of *Cyld*^–/–^ mice subjected to the TFC test.

### Reduced Intrinsic Excitability of the Principal Neurons in the Basolateral Amygdala of *Cyld*^–/–^ Mice

**FIGURE 3:**
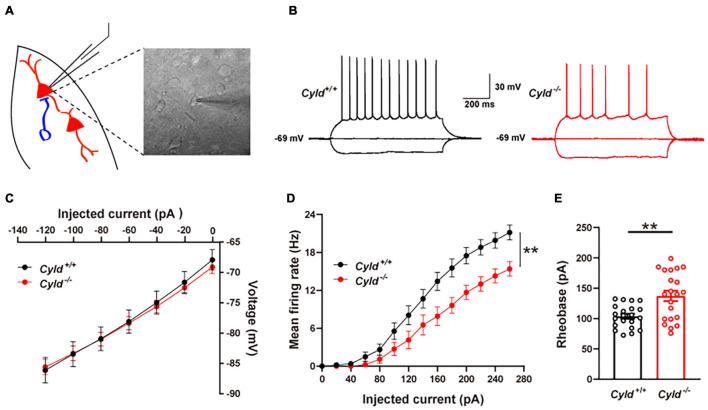
Reduced intrinsic excitability of the principal neurons in the BLA of *Cyld*^–/–^ mice. **(A)** Recording diagrams of the patch-clamp experiment. Pyramidal neurons in the BLA of acute brain slices from *Cyld*^–/–^ and *Cyld*^+/+^ mice were recorded in a whole-cell configuration. **(B)** Representative AP traces evoked by the hyperpolarized current (-120 pA) and depolarizing current (120 pA) injection. **(C)** Similar current–voltage characteristic curve of the principal neurons in the BLA of *Cyld*^–/–^ and *Cyld*^+/+^ mice. *n* = 21 neurons from seven mice for each genotype. **(D)** Principal neurons from the BLA of *Cyld*^–/–^ mice exhibited a lower number of APs in response to current injections ranging from 0 to 260 pA in steps of 20 pA. *n* = 21 neurons from seven mice for each genotype. **(E)** A higher rheobase current was needed to elicit an AP in *Cyld*^–/–^ mice. *n* = 21 neurons from seven mice for each genotype; Student’s *t*-test, **p < 0.01. Data are presented as mean ± SEM.

There are three possible causes of reduced neuronal activation following behavioral stimulation: decreased intrinsic excitability of neurons, depressed excitatory synaptic activity, or increased inhibitory synaptic activity (Sun et al., 2018). To explore the possible cellular mechanisms of aberrant neuronal activation in *Cyld*^–/–^ mice, we first examined whether CYLD regulates the neuronal excitability of principal neurons in a whole-cell configuration (Figure 3A). There are at least six types of interneurons in mouse BLA, whose firing patterns are different from principal neurons (Polepalli et al., 2020). Cells were abandoned when it exhibited any kinds of the firing patterns of interneurons. Representative AP traces are shown in Figure 3B. Electrophysiological properties including membrane capacitance, resting membrane potential, AP amplitude, AP half-width, AP rise time, AP decay time, after-hyperpolarization of AP, input resistance, and current–voltage curve of the principal neurons in the BLA of *Cyld*^–/–^ mice were comparable to those of *Cyld*^+/+^ mice (Table 1 and Figure 3C). However, *Cyld*^–/–^ mice showed a higher AP threshold [Table 1; Student’s *t*-test, *t*_(40)_ = 2.870, *p* = 0.007]. In addition, principal neurons from the BLA of *Cyld*^–/–^ mice exhibited a lower number of APs in response to current injections ranging from 0 to 260 pA (performed in 20 pA steps) [Figure 3D; two-way ANOVA_(genotype effect)_, *F*_(1,40)_ = 9.496, *p* = 0.0037] and a higher rheobase current [Figure 3E; Student’s *t*-test, *t*_(40)_ = 3.511, *p* = 0.0019] than principal neurons from *Cyld*^+/+^ littermates. Taken together, these data indicate that CYLD is required to maintain the excitability of principal neurons in the BLA.

**TABLE 1:** Intrinsic properties of the BLA principal neurons in *Cyld*^+/+^ and *Cyld*^–/–^ mice.

	***Cyld* ^+/^ *^+^***	***Cyld* ^–/–^**
	**(*n* = 19–21 neurons/7 mice)**	**(*n* = 19–21 neurons/7 mice)**
Membrane capacitance (pF)	92.413.82	90.344.62
Resting membrane potential (mV)	−67.881.70	−69.071.08
AP amplitude (mV)	73.062.03	75.201.75
AP half-width (ms)	1.140.04	1.270.06
AP rise time (ms)	0.350.02	0.380.02
AP decay time (ms)	0.820.04	0.950.05
After-hyperpolarization (mV)	−5.250.81	−4.410.96
Input resistance (MΩ)	160.2010.26	153.949.72
Threshold (mV)	−37.161.23	−31.941.34^**^

*Data are presented as the mean ± SEM.*

^**^p < 0.01, Student’s *t-*test.

### Depressed Excitatory Synaptic Activity in the Basolateral Amygdala of *Cyld*^–/–^ Mice

**FIGURE 4:**
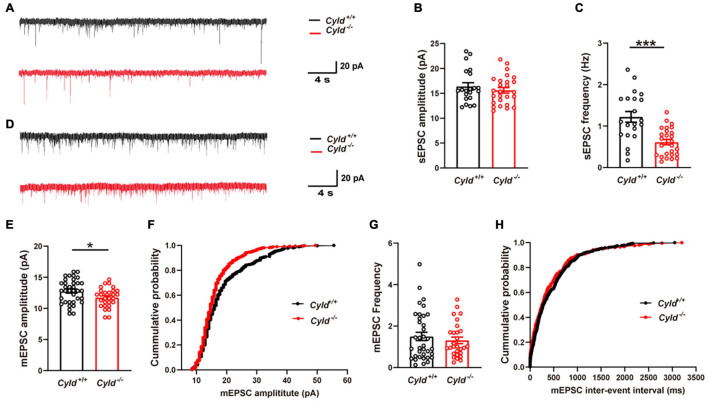
Compromised excitatory synaptic transmission in the BLA of *Cyld*^–/–^ mice. **(A)** Representative sEPSC traces continuously recorded from BLA principal neurons. **(B)** The sEPSC amplitude of principal neurons in the BLA was comparable between *Cyld*^+/+^ and *Cyld*^–/–^ mice. *n* = 26 neurons from seven mice for the *Cyld* knockout group, and *n* = 21 neurons from seven mice for the *Cyld*^+/+^ group. **(C)** Principal neurons in the BLA of *Cyld*^–/–^ mice showed a significant decrease in sEPSC frequency. *n* = 26 neurons from seven mice for the *Cyld* knockout group, and *n* = 21 neurons from seven mice for the *Cyld*^+/+^ group. **(D)** Representative mEPSC traces continuously recorded from BLA principal neurons. Average amplitude **(E)** and cumulative probability of amplitude **(F)** of mEPSCs in BLA principal neurons. *n* = 36 neurons from six mice for the *Cyld*^+/+^ group, and *n* = 28 neurons from four mice for the *Cyld* knockout group. Average frequency **(G)** and cumulative probability of the interevent intervals **(H)** of mEPSCs in BLA principal neurons. *n* = 36 neurons from six mice for the *Cyld*^+/+^ group, and *n* = 28 neurons from four mice for the *Cyld* knockout group; Student’s *t*-test, *p < 0.05, ***p < 0.001. Data are presented as mean ± SEM.

In view of the reduced excitability of principal neurons, we first examined whether AP-dependent sEPSCs of principal neurons were altered in the BLA of *Cyld*^–/–^ mice. Representative sEPSC traces are shown in Figure 4A. The sEPSC amplitudes of principal neurons in the BLA were comparable between *Cyld*^+/+^ and *Cyld*^–/–^ mice [Figure 4B; *Cyld*^+/+^: 16.4 ± 0.7 pA; *Cyld*^–/–^: 15.6 ± 0.6 pA; Student’s *t*-test, *t*_(45__)_ = 0.863, *p* = 0.3927]. However, consistent with their decreased neuronal excitability, we observed a significant decrease in the frequency of sEPSCs in principal neurons in the BLA of *Cyld*^–/–^ mice [Figure 4C; *Cyld*^+/+^: 1.2 ± 0.1 Hz; *Cyld*^–/–^: 0.6 ± 0.1 Hz; Student’s *t*-test, *t*_(45)_ = 4.447, *p* < 0.0001]. We next recorded mEPSCs in principal neurons of the BLA (Figure 4D). The data showed that mEPSC amplitude in principal neurons was lower in the BLA of *Cyld*^–/–^ mice [Figures 4E,F; *Cyld*^+/+^: 12.8 ± 0.3 pA; *Cyld*^–/–^: 11.7 ± 0.3 pA; Student’s *t*-test, *t*_(62__)_ = 2.312, *p* = 0.0241]. However, the mEPSC frequency was not affected by the mouse genotype [Figures 4G,H; *Cyld*^+/+^: 1.5 ± 0.2 Hz; *Cyld*^–/–^: 1.3 ± 0.2 Hz; Student’s *t*-test, *t*_(62)_ = 0.722, *p* = 0.4729]. Together, these results indicate that *Cyld* mutation alters excitatory synaptic transmission in the BLA. This may be an underlying mechanism responsible for the aberrant neuronal activation in *Cyld*^–/–^ mice.

### Increased Inhibitory Synaptic Activity and Expression of Parvalbumin in the Basolateral Amygdala of *Cyld*^–/–^ Mice

**FIGURE 5:**
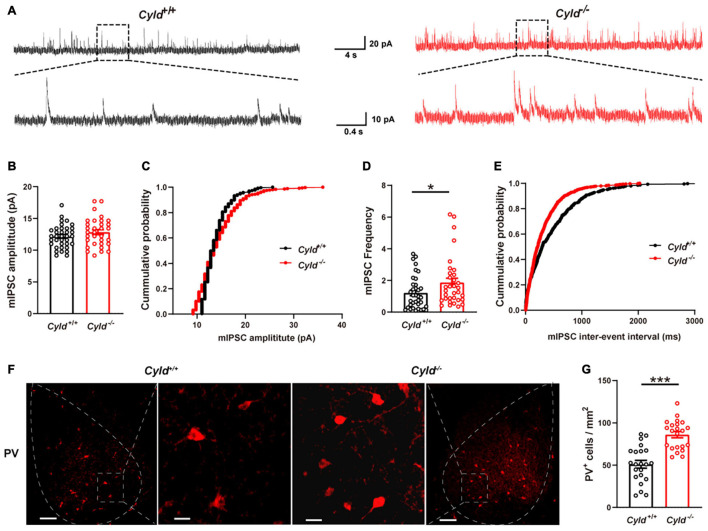
Excessive inhibitory activity in the BLA of *Cyld*^–/–^ mice. **(A)** Representative mIPSC traces continuously recorded from BLA principal neurons. Average amplitude **(B)** and cumulative probability of amplitude **(C)** of mIPSCs in BLA principal neurons. *n* = 36 neurons from six mice for the WT group, and *n* = 32 neurons from four mice for the *Cyld* knockout group. Average frequency **(D)** and cumulative probability of the interevent intervals **(E)** of mIPSCs in BLA principal neurons. *n* = 36 neurons from six mice for the *Cyld*^+/+^ group, and *n* = 33 neurons from four mice for the *Cyld* knockout group. **(F)** Representative images of parvalbumin-expressing interneurons in the BLA of *Cyld*^+/+^ and *Cyld*^–/–^ mice. Scale bar: 100 μm. For enlarged images of areas in dotted square, scale bar = 20 μm. **(G)** Quantitative analysis showed that the number of PV^+^ neurons in BLA was significantly increased. *n* = 22 slices from eight mice for each group. Student’s *t*-test, *p < 0.05, ***p < 0.001. Data are presented as mean ± SEM.

Reduced neuronal activation upon behavioral stimulation could also be due to increased inhibitory synaptic activity (Sun et al., 2018). To investigate this, we examined mIPSCs in principal neurons in the BLA. Representative mIPSC traces are shown in Figure 5A. As shown in Figures 5B,C, mIPSC amplitude was not altered in the BLA of *Cyld*^–/–^ mice [*Cyld*^+/+^: 12.2 ± 0.3 pA; *Cyld*^–/–^: 12.8 ± 0.4 pA; Student’s *t*-test, *t*_(66__)_ = 1.369, *p* = 0.1757]. However, an increase in mIPSC frequency in principal neurons was observed in the BLA of *Cyld*^–/–^ mice [Figures 5D,E; *Cyld*^+/+^: 1.2 ± 0.2 Hz; *Cyld*^–/–^: 1.9 ± 0.3 Hz; Student’s *t*-test, *t*_(67__)_ = 2.023, *p* = 0.0471]. This result suggests that CYLD plays a role in regulating inhibitory synaptic transmission and may therefore have an effect on neuronal activation in the BLA of mice upon behavioral stimulation.

The presence of PV, a calcium-binding protein, is characteristic of fast-spiking interneurons (Trantham-Davidson and Lavin, 2019). PV^+^ interneurons act as a feedforward inhibitor to regulate AP initiation in principal neurons (Hu et al., 2014) and likely contribute to the inhibitory synaptic transmission (Wang et al., 2020). To further explore the possible mechanism of the reduced intrinsic excitability and aberrant inhibitory synaptic transmission we observed in the principal neurons of *Cyld*^–/–^ mice, we conducted immunohistochemical staining to determine the number of PV^+^ interneurons in the BLA (Figure 5F). Data analysis revealed a significant increase in the number of PV^+^ interneurons in the BLA of *Cyld*^–/–^ mice compared with WT [Figure 5G; 51.0 ± 4.6 and 86.0 ± 3.7 cells/mm^2^ for *Cyld*^+/+^ and *Cyld*^–/–^ mice, respectively; Student’s *t*-test, *t*_(42__)_ = 5.916, *p* < 0.0001]. Taken together, these results suggest that CYLD is required to maintain the number of PV^+^ interneurons in the mouse BLA. Moreover, the reduced intrinsic excitability and increased frequency of mIPSCs in the BLA of *Cyld*^–/–^ mice described previously may relate to the increased number of PV^+^ interneurons.

## Discussion

Here, for the first time, we show that the K63-deubiquitinase (DUB), CYLD, plays an important role in modulating fear memory. The gene that encodes CYLD is highly expressed in the striatum followed by the NAc, amygdala, hypothalamus, PFC, and hippocampus (see text footnote 1). CYLD has been studied extensively for its role in negatively regulating NF-κB signaling (Lork et al., 2017), but its function in mammalian brain remains largely unknown. Previous proteomic analyses using mouse or rat brain samples indicated the presence of CYLD in purified PSD (Li et al., 2019). Other studies revealed that CYLD regulates dendritic growth and postsynaptic spine maturation (Ma et al., 2017; Li et al., 2019). What these findings imply is that CYLD may regulate learning and memory of mammalian brain.

Appropriate fear memory in response to potential danger in the environment is crucial for animal survival, while abnormal fear memory is implicated in several mental disorders (Nielen et al., 2009; Bowers and Ressler, 2015; Taylor and Torregrossa, 2015; Varinthra et al., 2021). The BLA is an obvious region of interest in view of its central role in the fear circuitry, mediating both fear learning and its expression (Luyck et al., 2020). Our TFC test revealed that *Cyld* knockout impairs fear memory in mice. Recent studies in which researchers performed whole-exome sequencing in FTD patients also showed that patients carrying *Cyld* mutations suffer severe memory impairment (Dobson-Stone et al., 2020; Tábuas-Pereira et al., 2020). In addition, numerous studies have revealed the role of other DUBs (USP2, USP6, USP14, USP46, and UCHL1) in promoting learning and memory (recognition memory, fear memory, and spatial memory) in rodents (Imai et al., 2013; Jarome et al., 2013; Zhang et al., 2014; Zeng et al., 2019; Srikanta et al., 2021). These reports indicate that DUBs including CYLD are involved in fear memory modulation.

There are two necessary detection units for tone-cued fear conditioning: an auditory sensation-based cue and noxious cutaneous stimuli (Silva et al., 2016). Thus, the auditory sensation and nociception of mice are essential physiological factors to form the fear memory. In our study, all mice showed a similar response to the presentation of the tone during the training period, indicating that *Cyld* knockout mice did not experience tone-cued fear conditioning-related sensory encoding dysfunctions. We also consider other factors that may affect the fear behavior. A previous report showed that *Cyld*^–/–^ mice exhibited normal motor function and did not show depression-like behavior (Han et al., 2020). However, anxiety-like behavior was found in *Cyld*^–/–^ mice (Han et al., 2020). In the current study, *Cyld*^–/–^ and *Cyld*^+/+^ mice showed a similar freezing response during the habituation and pre-tone period, thus excluding the influence of anxiety. Therefore, the low freezing level that we observed in *Cyld*^–/–^ mice during the tone-cued test could be regarded as a fear memory deficit.

The formation of fear memory comprises three phases: acquisition [short-term memory (STM)], consolidation (STM is stabilized into a persistent long-term memory), and reconsolidation (long-term memory) (Johansen et al., 2011). Interestingly, we found that *Cyld* knockout had no effect on the acquisition phase of fear memory. One likely reason could be that plasticity is important for immediate learning and that STM is mediated by covalent modification of existing synaptic proteins (for example, by phosphorylating glutamate receptors) (Johansen et al., 2011). In contrast, consolidation and reconsolidation of this plasticity are generally thought to occur *via* activation of second messengers that initiate gene transcription and translation of new proteins like activity-regulated cytoskeletal-associated proteins and early growth response protein 1 (Johansen et al., 2011). CYLD may not affect existing synaptic proteins in the short-term, but instead may play an important role in long-term modulation of consolidation- and reconsolidation-related protein synthesis.

c-Fos is extensively used as a marker of neuronal activation in the BLA during the response to external stimuli such as fear learning, fear conditioning with an auditory cue, and memory retrieval (Knapska et al., 2007). Previous reports showed increased expression of c-Fos in the BLA in response to fear conditioning (Rajbhandari et al., 2016; Sun et al., 2018; Silva et al., 2019). Here, we found that the number of c-Fos^+^ neurons in response to the tone-cued test is reduced in the BLA of *Cyld*^–/–^ mice. However, the number of c-Fos^+^ neurons of *Cyld*^+/+^ mice was comparable to that of *Cyld*^–/–^ mice, indicating an effect of CYLD on fear memory-related neuronal activation. Interestingly, statistical differences of the number of c-Fos^+^ neurons between *Cyld*^+/+^ and *Cyld*^–/–^ mice were not found in the tone-only test or pre-tone stage. Together with the behavioral results, our study shows that CYLD is critical for mice to associate the CS and US during the fear memory retrieval. Generally, fear memory impairment may associate with the aberrant neuronal activation in the amygdala of mice (Huang et al., 2014; Sun et al., 2018). A reduction in neuronal activation upon behavioral stimulation could be caused by decreased intrinsic excitability of neurons, depressed excitatory synaptic activity, or increased inhibitory synaptic activity (Sun et al., 2018). It is well established that excitation of principal neurons in the BLA is a critical step in auditory fear conditioning (Johansen et al., 2010; Yiu et al., 2014; Polepalli et al., 2020). Coincident with the fear memory impairment and aberrant neuronal activation we observed, there was also an increase in the threshold and rheobase of APs and a decrease in the AP firing rate of *Cyld*^–/–^ mice, which suggests reduced neuronal excitability in BLA principal neurons. Another study also showed that the cellular mechanisms underlying changes in fear memory involve modulation of intrinsic excitability of neurons (Zhang et al., 2017).

Among K63-DUBs localized in the PSD, CYLD emerges as a central player (Zajicek and Yao, 2021). The abundance of CYLD in the PSD suggests that it may be the primary DUB for multiple proteins involved in synapse development, function, and plasticity, as well as related pathologies (Ma et al., 2017). Therefore, we examined the role of CYLD in modulating synaptic transmission in the BLA to further explore the possible cellular mechanisms of aberrant neuronal activation in *Cyld*^–/–^ mice. In line with the reduced AP firing rate, we observed a significant decrease in the sEPSC frequency of principal neurons in the BLA of *Cyld*^–/–^ mice. In addition, *Cyld* knockout reduces the mEPSC amplitude of these principal neurons. Our results suggest that CYLD plays an important role in modulating excitatory synaptic transmission and the levels of postsynaptic α-amino-3-hydroxy-5-methyl-4-isoxazole propionic acid receptors (AMPARs) or NMDA receptors. K63-linked ubiquitination chains are considered the primary non-degradable linkage, and these chains can also remodel the surface of substrate proteins, akin to phosphorylation, to directly regulate protein–protein interactions (Ma et al., 2017). Therefore, CYLD may be involved in the synthesis, turnover, and trafficking of proteins in the PSD. These functions of CYLD were implied in a study on another K63-DUB, USP46, which can cleave K63-linked ubiquitination chains from AMPARs and modulate AMPAR internalization and turnover in cultured rat cortical and hippocampal neurons; in this way, USP46 is thought to regulate AMPAR-mediated excitatory synaptic transmission (Huo et al., 2015). A recent study showed that CYLD can cleave K63-linked ubiquitination chains from PSD-95 (Ma et al., 2017), which represents the first direct evidence that CYLD modulates postsynaptic scaffolding and remodeling in excitatory synapses.

In view of the impaired excitatory synaptic transmission in the BLA of *Cyld*^–/–^ mice, we also recorded mIPSCs in BLA principal neurons. It turned out that *Cyld* mutation increased mIPSC frequency, but had no effect on mIPSC amplitude. This result implies the possibility of a presynaptic change in the release of inhibitory neurotransmitters or the number of GABAergic interneurons. A large body of evidence suggested that the formation and expression of conditioned fear memories also entails long-lasting functional and structural plasticity of GABAergic synapses onto pyramidal neurons of the murine BLA (Kasugai et al., 2019). Available data support the contention that PV^+^ interneurons play an important role in modulating the balance of excitation and inhibition in local circuits and they are thus involved in the regulation of fear memory (Hu et al., 2014; Lucas and Clem, 2018; Wang et al., 2020). In addition, PV^+^ interneurons form synaptic contacts predominantly onto the soma as well as proximal axon and dendrites of the principal neurons in BLA which allow them to modulate the rate and timing of the principal neuron spike initiation (Woodruff and Sah, 2007; Hu et al., 2014; Lucas and Clem, 2018). Hence, we examined PV^+^ interneurons, finding that the number of BLA PV^+^ interneurons was significantly higher in *Cyld*^–/–^ mice, consistent with excessive local circuit inhibition. This result suggests that increased number of PV^+^ interneurons in BLA of *Cyld*^–/–^ mice may disrupt the balance of excitation and inhibition in local circuits which could be associated with the fear memory deficit observed in *Cyld* mutant mice. A previous study also showed that *Cyld* mutation causes an excess of striatal GABAergic inhibition (Zhang et al., 2016). In addition, loss of the DUB, USP14, reduced neurotransmitter release and increased surface expression of the GABA_A_R (Jarome et al., 2013). Taken together, these data suggest that CYLD is also crucial for maintaining inhibitory synaptic transmission in the BLA.

Research on CYLD is still at an early stage. Recent studies have focused on the role of CYLD in regulating synaptic plasticity. The abundance of CYLD in the PSD suggests that it may be the primary DUB for multiple proteins (Ma et al., 2017). More studies are needed to identify and characterize these target proteins, because this could provide insights into the mechanisms of synapse function and plasticity. Our study indicates that CYLD may provide a novel target for therapeutic interventions for various fear memory-associated brain disorders. Except for being expressed in the BLA, *Cyld* is also expressed in several brain regions like the striatum, NAc, CeA, and PFC (see text footnote 1), which are also involved in fear memory. It has long been known that the PFC and CeA are tightly involved in tone-cued fear memory (Ferreira et al., 2008; Tronson et al., 2012; Duvarci and Pare, 2014; Marek et al., 2019). In addition, a previous study showed that the flow of information between the basal amygdala and the NAc is necessary for signaled active avoidance behavior (Ramirez et al., 2015). It is worth noting that the indirect CeA–dorsal striatum pathway mediates tone-cued fear memory (Ferreira et al., 2008). Thus, we cannot exclude the possibility that CYLD deficiency in brain regions mentioned above are the source of the impaired fear memory of *Cyld*^–/–^ mice. Specifically ablating the *Cyld* of the principal neurons in BLA and observing the consequent fear memory, as well as the electrophysiological outcomes, are required to establish the precise link between CYLD in BLA and tone-cued fear memory. In addition, chemogenetically operating the principal neurons in BLA of conditional *Cyld* knockout mice would help to further elucidate the linkage mentioned above.

## Data Availability Statement

The raw data supporting the conclusions of this article will be made available by the authors, without undue reservation.

## Ethics Statement

The animal study was reviewed and approved by the Ethics Committee of Animal Research of South China Normal University.

## Author Contributions

H-dL designed and performed electrophysiological, immunostaining, and behavioral experiments, analyzed the data, and wrote the manuscript. D-nL performed part of the fear conditioning behavioral test, immunostaining experiments, and data analysis. LY provided suggestions on experimental design and improvement of the manuscript. CL supervised the study, designed the experiments, and made critical revision of the manuscript. All authors contributed to the article and approved the submitted version.

## Conflict of Interest

The authors declare that the research was conducted in the absence of any commercial or financial relationships that could be construed as a potential conflict of interest.

## Publisher’s Note

All claims expressed in this article are solely those of the authors and do not necessarily represent those of their affiliated organizations, or those of the publisher, the editors and the reviewers. Any product that may be evaluated in this article, or claim that may be made by its manufacturer, is not guaranteed or endorsed by the publisher.

## Footnotes

1
www.biogps.org

2
mouse.brain-map.org
